# Lessons learned from implementation of computerized provider order entry in 5 community hospitals: a qualitative study

**DOI:** 10.1186/1472-6947-13-67

**Published:** 2013-06-24

**Authors:** Steven R Simon, Carol A Keohane, Mary Amato, Michael Coffey, Bismarck Cadet, Eyal Zimlichman, David W Bates

**Affiliations:** 1 VA Boston Healthcare System, Boston, MA, USA; 2 Brigham and Women’s Hospital, Boston, MA, USA; 3 Massachusetts College of Pharmacy and Health Sciences-Boston, Boston, MA, USA; 4 North Shore Physicians Group, Saugus, MA, USA; 5 Winchester Hospital, Winchester, MA, USA

**Keywords:** Quality of care, Clinical decision support, Meaningful use, Transformation

## Abstract

**Background:**

Computerized Provider Order Entry (CPOE) can improve patient safety, quality and efficiency, but hospitals face a host of barriers to adopting CPOE, ranging from resistance among physicians to the cost of the systems. In response to the incentives for meaningful use of health information technology and other market forces, hospitals in the United States are increasingly moving toward the adoption of CPOE. The purpose of this study was to characterize the experiences of hospitals that have successfully implemented CPOE.

**Methods:**

We used a qualitative approach to observe clinical activities and capture the experiences of physicians, nurses, pharmacists and administrators at five community hospitals in Massachusetts (USA) that adopted CPOE in the past few years. We conducted formal, structured observations of care processes in diverse inpatient settings within each of the hospitals and completed in-depth, semi-structured interviews with clinicians and staff by telephone. After transcribing the audiorecorded interviews, we analyzed the content of the transcripts iteratively, guided by principles of the Immersion and Crystallization analytic approach. Our objective was to identify attitudes, behaviors and experiences that would constitute useful lessons for other hospitals embarking on CPOE implementation.

**Results:**

Analysis of observations and interviews resulted in findings about the CPOE implementation process in five domains: governance, preparation, support, perceptions and consequences. Successful institutions implemented clear organizational decision-making mechanisms that involved clinicians (governance). They anticipated the need for education and training of a wide range of users (preparation). These hospitals deployed ample human resources for live, in-person training and support during implementation. Successful implementation hinged on the ability of clinical leaders to address and manage perceptions and the fear of change. Implementation proceeded smoothly when institutions identified and anticipated the consequences of the change.

**Conclusions:**

The lessons learned in the five domains identified in this study may be useful for other community hospitals embarking on CPOE adoption.

## Background

Computerized provider order entry (CPOE), especially when coupled with clinical decision support (CDS), can lead to improved safety, quality and efficiency of care, by preventing medication errors, avoiding redundant testing and promoting the use of evidence-based treatments, among other mechanisms [1-5]. Although most studies and systematic reviews have suggested that CPOE results in better, safer care, other reports have questioned the magnitude of CPOE’s effects and the generalizability of findings from the limited number of academic centers that have adopted CPOE [6,7]. Despite these potential benefits, and the wide availability of commercially developed CPOE systems for nearly two decades, most hospitals in the United States do not have CPOE [8]. Hospitals face a host of barriers to adopting CPOE, ranging from resistance among physicians to the cost of the systems.

The Health Information Technology for Economic and Clinical Health (HITECH) Act, [9] which will award multi-million dollar incentive payments to hospitals that can demonstrate meaningful use of CPOE, represents a major policy shift that has already begun to accelerate US hospitals’ adoption of CPOE [10]. Because so few hospitals have experience with implementing CPOE, and because many of the early adopters of CPOE were academic medical centers and/or hospitals that engineered homegrown CPOE systems, community hospitals embarking on the implementation of commercial CPOE systems have little accumulated experience to guide their approach. Furthermore, few studies have been done of vendor or community hospital implementation, and their experiences may differ in many ways from academic hospitals.

In Massachusetts, five community hospitals agreed to participate in a before-after study of CPOE implementation in 2005. From 2006 to 2009, these hospitals implemented commercially-purchased CPOE systems. In this study, we used qualitative research methods to characterize the experience of hospital staff – including nurses, physicians, pharmacists and hospital leaders – relative to CPOE implementation, with the goal of identifying generalizable lessons that could help to guide other community hospitals undertaking CPOE implementation.

## Methods

### Study design

We designed a qualitative study to capture various perspectives of the CPOE implementation process in five community hospitals. The methods included site visits for field observation and in-depth interviews with key informants at all five facilities. The study protocol and instrument materials were reviewed by the Partners Healthcare Human Research Committee and by the study site committees and determined to meet criteria for exemption from further IRB review per the regulations found at 45 CFR 46.101(b) (2) Use of educational tests (cognitive, diagnostic, aptitude, achievement), survey procedures, interview procedures, or observation of public behavior. The authors attest that the study was conducted in compliance with the World Medical Association’s Declaration of Helsinki - Ethical Principles for Medical Research Involving Human Subjects.

### Setting

The five study sites were among six hospitals that agreed to participate in a pre-post evaluation of CPOE in 2005 [11,12]. Study sites volunteered to participate and were representative of small to medium-sized hospitals in Massachusetts. One hospital subsequently decided not to implement CPOE and was therefore not included in this study. Each hospital had 100–300 inpatient beds; two facilities had house staff (i.e., resident physicians-in-training), and three did not.

### Site visits

Drawing on the published literature, [13,14] we developed an instrument for field observation of the current state of CPOE adoption and use in inpatient settings. The instrument guided the observer to describe the range of individuals and behaviors involved in the ordering of clinical interventions (e.g., medication prescriptions, laboratory testing) on the inpatient units of each hospital. The guide prompted the observer to record both structured data (e.g., “What proportion of orders are entered directly by physicians via CPOE?”) and unstructured information (e.g., “Describe the typical workflow of this setting.”). Three research nurses, all with inpatient clinical experience as well as experience abstracting medical records for research, completed the site visits. Each of the sites was observed for a minimum of 8 hours; the research nurses attempted to observe multiple clinical units (e.g., medical-surgical floor, ICU) at each facility. The nurses were encouraged to write field notes to summarize their observations following each site visit.

### In-depth interview guide

We developed an interview guide consisting of open-ended questions to explore how the study participants perceived the process of CPOE implementation. The guide included core questions that covered the following topics: attitudes toward CPOE prior to and at the time of its implementation; barriers to implementation; factors that facilitated implementation; current goals for advancing the use of CPOE and potential impediments to doing so; advice for other hospitals considering or embarking on CPOE implementation. The interviewer used this list of core questions, adding follow-up probes as necessary to clarify relevant avenues of inquiry raised by the participants.

### Participants

Our original sampling strategy for the in-depth interviews was to identify a minimum of five individuals at each facility, including at least one clinician who orders treatments through CPOE (e.g., physician, nurse practitioner or physician assistant), at least one professional who processes clinician-requested orders (i.e., nurse or pharmacist), and at least one hospital executive with oversight of the CPOE implementation process. Each site’s study steering committee provided a list of potential interviewees, whom we contacted by telephone to recruit for the study. We asked the steering committee to suggest individuals who would be representative of their peers and who would be likely to speak with candor. Although some physicians were unable to participate because of scheduling conflicts, no individuals refused to participate. None of the participants had a relationship with the interviewer prior to the study. We completed 24 in-depth interviews (Table 1).

**Table 1:** Distribution of 24 participants in in-depth interviews at 5 community hospitals

**Site**	**Physicians interviewed (N)**	**Nurses interviewed (N)**	**Pharmacists interviewed (N)**
Hospital 1	2	4	0
Hospital 2	1	2	1
Hospital 3	3	2	1
Hospital 4	2	2	1
Hospital 5	1	2	0
**Total**	**9**	**12**	**3**

### Data collection

Interviews were conducted by study investigators (SRS, a male physician; CAK, a female registered nurse; MA, a female pharmacist; and EZ, a male physician) with experience in in-depth interviewing for qualitative research. All interviews were conducted by telephone, audio recorded and transcribed. Interviews and site visits were conducted May-December, 2010.

### Data analysis

The inter-disciplinary project analysis team consisted of physicians (SRS, MC, BC, EZ) a nurse (CAK) and a pharmacist (MA). The team conducted a group method of content analysis known as immersion/crystallization [15]. Team members independently listened to selected interview tapes, read all transcripts and wrote analytic notes. The team members then met regularly to discuss the transcripts. Detailed notes were typed during these meetings to document emerging themes and maintain a permanent record of our analysis progress. This process allowed us to become thoroughly immersed in the data within the context of each transcript in its entirety, and we were able to compare the data from the transcript under discussion with the data from other analyzed transcripts. Next, we created a spreadsheet for including salient data and illustrative quotes from each interview transcript for barriers to implementation and facilitators of implementation at each of the 5 study sites, arrayed by participants’ role in the hospital (i.e., ordering clinician; nurse or pharmacist; executive). This combined procedure allowed us to compare and contrast themes within and between hospitals as well as within and between professional roles.

## Results

### Dominant content areas

In the analysis of the 24 interviews and observations of the 5 study settings, five themes emerged as having particular relevance to the process of CPOE implementation: Governance, Preparation, Support, Perceptions and Consequences. The lessons learned from these five domains, along with representative quotations from participants, are summarized in Table 2.

**Table 2:** Lessons learned from CPOE implementation in five community hospitals*

**Lesson**	**Action**	**Representative quotation**
*Lesson 1*: Governance Matters	Establish a clear organizational decision-making mechanism and involve clinicians in it.	“We talk about success factors. I think that the principal one was to have all the key stakeholders sitting at the table together to make decisions relative to CPOE…. Another core piece was that we did have a physician champion who was leading the effort and serving as liaison between the IS department and the physician community. I will have to say that the IS (Information Systems) department did a lot of heavy lifting in all of this, which was okay with us, as long as the other people were participating in the process.” (Registered Nurse)
*Lesson 2*: Preparation and Advance Planning	Expect the need for multiple methods of training, including the most basic computer skills for novice users.	“It was a huge training process. I think that it was a two-day class that they went to train you how to use it." (Registered Nurse)
*Lesson 3*: Support	Deploy highly trained peer users to provide live, in-person, "at-the-elbow" support during the immediate go-live period.	“Subject matter experts were available on our inpatient wards early on in the implementation phase. So, if people were struggling with an order, they wouldn’t have to call somebody on the phone. There would be somebody there to help them.” (Physician)
*Lesson 4*: Managing Perceptions	Encourage strong clinical leaders to address the fear of change.	“Yeah, people were afraid. They were afraid of the change and if things happened, if the computer went down, you’d lose your information – not realizing there were backups.” (Administrator)
*Lesson 5*: Consequences	Anticipate consequences and have a process to address them.	“CPOE has absolutely made the order system easier. It has expedited care. I think it’s a big benefit as far as, you know, patient safety. I think that compared with telephone orders, requiring physicians physically to enter their orders in the system, this has improved the speed at which we are able to get things done around here. That being said, I also feel a potential issue is that physicians are able to make changes without communicating directly with the nurses. So, sometimes there will be orders being entered while the nurses are away from the computer system and we are not aware of it, so orders that may be urgent or the physician may want our attention on, there’s a lag time until the time the nurse actually sees the order, versus the physician speaks to the nurses directly. And the system makes that really easy (to occur), because you don’t have to be in the unit to deal with the chart, and they (physicians) don’t have to call the unit to speak with the nurse. So, there’s a little bit of communication breakdown.” (Registered Nurse)

*See text for detailed explanation and additional examples of each lesson.

### Governance matters

While none of the interviewees used the word “governance”, all hospital leaders, and a majority of the physicians, pharmacists and nurses, alluded to the importance of thoughtful composition and successful functioning of committees and working groups to establish goals, timelines and policies. All emphasized the importance of multi-disciplinary representation and collaboration. In particular, participants from all five sites reported that having front line (physician, nurse, pharmacist) representation on committees charged with establishing policies was essential to ensuring realistic approaches that would be acceptable to the clinical staff and dealing with problems that arose. “The departments that were most affected (by CPOE implementation) were nursing and physicians, who were the front-end users, so to speak. As a result, we (physicians and nurses) were the bulk of the people at the table.” (Nurse Administrator)

### Preparation and advance planning

Preparation and advance planning for CPOE implementation were emphasized across all five sites by physicians, nurses, pharmacists and the leaders of the organizations. This preparation took various forms, ranging from planning meetings among implementation work groups to organized training sessions for staff in basic computer skills as well as in the use of the CPOE system itself:

• Interviewer: Tell me what you remember about the weeks leading up to implementation.

• Nurse Manager: Weeks? It was actually months leading up. We had a core team develop a multi-disciplinary team from across the system and we met weekly for two hours. We had the help of a (full-time) project coordinator hired by IT, who was phenomenal.

All five hospitals arranged for training sessions for their staff, but opinion varied as to how valuable these sessions actually were:

“As you might imagine, you can do as much didactic, even virtual, training at computers, which is what we did. But until you start to use the product ‘live’, that’s when more of the questions and concerns arose.” (Physician)

The majority of physician, nurse and pharmacist respondents from all sites felt that their hospital’s efforts to prepare staff were sufficient, with a few notable exceptions. One senior nurse executive commented that the physicians on staff were not sufficiently prepared for the transition: “I think they were just not prepared. They didn’t realize just how challenging navigating the IT was going to be.” (Registered Nurse) This perception contrasts with a nurse at a different site, who described herself as “very slow using the computer,” who felt very well prepared by the hospital: “The hospital told you about it ahead of time, what was going to go on. They had those training sessions. And during a month or so, they had the super users and stuff. I think it just worked out very nicely. They had the whole package how to train the people, for us to be able to use it. It made the transition much easier.” (Registered Nurse)

In thinking about the role of training as preparation for CPOE implementation, many nurses and physicians naturally shifted the conversation to one of two topics. In some cases, the interviewees emphasized that advance training was useful, but real-time support was far more important. In other cases, the nurses and physicians characterized their institution’s preparation for implementation in the context of understanding and “managing” the prevailing perceptions. These topics – support and perceptions – are discussed below.

### Support

Perhaps the most consistent factor cited as helpful in facilitating the transition to CPOE implementation was the ample presence of live, in-person support – usually from specially trained nurses, physicians, or pharmacists labeled as “superusers” – during the period immediately following the “go-live” date. “Even after it (CPOE) got implemented on the floor, they had all these superusers who were around constantly in case you had any question. That probably went on for almost a month.” (Registered Nurse)

Physicians and nurses at one hospital referred to this kind of support as “at the elbow,” a term used in the health information technology industry to mean the kind of in-person, one-on-one support, usually provided by peers with expertise, during the course of actual care delivery when an individual is struggling to use a system [16,17]. Several nurses pointed out a countervailing perspective on the role of at-the-elbow “superusers” and the importance of having sufficient support – operationalized here as “manpower.” These nurses pointed out that in their hospital, the superusers were specially trained medical-surgical nurses who had expressed interest in providing support for CPOE implementation. These nurse-superusers became unavailable for “regular” staff assignments. “It was frustrating that staff members were taken out of the rotation to work as a superuser for two weeks at a time. We ended up using overtime and dipping into our pool of ‘float’ nurses.” (Registered Nurse)

### Perceptions

Managing the prevailing perceptions of the institution emerged as an integral element to the success of implementation. In this context, “perceptions” refers to the attitudes and mood of the individuals and groups of individuals in each setting prior to and following CPOE implementation. At all five hospitals, every nurse, pharmacist and physician acknowledged the presence of some anxiety before implementation. “There was a lot of anxiety, from both physicians and the nursing staff…. There was concern about how this was going to change patient care, just the overall change in practice after so many years.” (Registered Nurse) Occasionally, the level of anxiety rose to fear: “I remember that there was a lot of angst with the physicians around jumping into CPOE and starting in with the new technology…. I would say fear.” (Registered Nurse)

When pushed to identify the source of the anxiety, the most common response, regardless of site or professional role, was the fear of change, rather than any specific concern about computers or the new system’s features: “There was a little anxiety. It’s the fear of change and that sort of thing.” (Registered Nurse)

One physician described even more intense fear and resistance: “There was tremendous pushback by a few doctors early on who swore this was going to make care more dangerous, who swore that docs were going to rebel. There were a couple of cardiologists … one who viewed himself as an IS (information systems) guru, who just swore that this was going to create pandemonium in the medical center and the doctors would rebel. And they wouldn’t use it and they found themselves on a bully pulpit early on with people standing around listening. And what happened was that fewer and fewer people were at the bully pulpit, until they were standing there by themselves.” This physician emphasized the role of leadership in overcoming these perceptions and attitudes: “It had a lot to do with our chief who just simply said, ‘We’re going to do this, and it’s better for patient care, and shut up and do it.’” (Physician)

An important observation that was made consistently across all five sites was that the fear and anxiety were generally more prevalent among older, more senior individuals. “I think there were two factions. From our older, more tenured population, fear, outright fear. These are people who are not computer savvy. That generation – they were frightened to death. But the converse of that was that our younger generation, they were thrilled, couldn’t be happier because the computer savvy person understands the benefit of technology. So, you really had to gear your education differently to those different groups and understand that you had to build in extra time for people who didn’t have the background.” (Nurse Administrator)

It is also worth noting that the anxiety and fear, common among nurses and physicians, were generally not noted among pharmacists. “I don’t recall there being tremendous amounts of anxiety on the (pharmacist) users. think a lot of them were looking forward to more technology and a safer practice.” (Pharmacy Informatics Manager) A pharmacy director noted, “(There was) definitely no resentment, for pharmacy our long struggle has been well documented with just the reading of handwriting. So, anything that was going to clean up the handwriting, we were all for it.”

### Consequences

Interviewees provided insights into consequences of CPOE implementation from two perspectives. Looking back, they described anticipated consequences and how those anticipations influenced implementation. As suggested above, at each hospital there existed some degree of anxiety or fear about CPOE implementation. This anxiety was often described generically as “fear of the unknown” but was also frequently ascribed to anticipated adverse consequences, specifically increased burden of work on the individual user (reported most frequently by physicians), computer usage leading to decreased time at the bedside (reported by nurses) or more generally in the clinical units (reported by pharmacists). Counterbalancing these fears was the expectation that CPOE would improve patient safety. Interviewees almost uniformly indicated that they and their peers anticipated that CPOE would reduce errors, citing most frequently the anticipated improved legibility of orders. Notably, physician champions and hospital leaders leveraged this vision of a safer system to motivate staff to embrace CPOE adoption, emphasizing the importance of safety instead of the potential financial benefits to be reaped.

Study participants frequently reflected back on what consequences they anticipated before implementation and the extent to which those consequences materialized. Throughout all of the interviews we conducted, no individual expressed a desire to return to a paper system of order entry. Nurses and pharmacists cited improved legibility of physicians’ orders as one of the most palpable and immediate beneficial consequences of CPOE implementation: “Before CPOE, a lot of times the doctor’s writing wasn’t very legible, so we would be calling them a lot. Now that everything is in the computer, that makes it much easier, because obviously you can read it. There are not as many questions.” (Registered Nurse)

While acknowledging the obvious safety and efficiency benefits of the CPOE system, nurses and physicians were equally aware of the unintended consequences of the new system. One of the most notable concerns voiced by nurses at two of the hospitals was the presence of a dual or hybrid system in which some physicians were placing orders within the CPOE system, while others were still writing paper orders. Nurses explained that they worried constantly that important patient information would get lost with the hybrid system. “And I think nursing had sort of a mixed sense because being in a quasi-CPOE environment was a challenge and the fact that some doctors used it and some didn’t sometimes created more problems than it solved.” (Registered Nurse)

With respect to safety, physicians recognized both the potential benefits of CPOE as well as its potential risks. One physician spoke at length:

“In terms of error rates, I have not noticed an improvement.” He went on to elaborate: “It’s much easier – I’ve heard this in public, and I’m sure it’s a common theme – where people order on the wrong patient. That is much easier to do on the computer system than in the paper and ink chart. I think it is probably not a problem for the younger generations, but I found it with me and other doctors that are using CPOE: we miss more things in some ways because you don’t see a list of it, where you can go through it easily…. On the med interactions, we get so many pop-ups that I think everyone ignores them…. It’s the alert fatigue. It’s just huge. There are many times you get an alert when you order aspirin. So I’ve been disappointed in terms of that.” (Physician)

We asked participants whether the implementation of CPOE led individuals – nurses, pharmacists, or physicians – to leave the institution. Responses were highly variable, with some respondents – regardless of site or professional role – expressing a strong belief that CPOE implementation did not result in any staff departures. In contrast, some interviewees noted that the CPOE implementation was a watershed moment for their hospital, accelerating the retirement of some senior nurses and changing the practice of some physicians: “As a result, many of them – it was actually a turning point for us – many of them decided that they weren’t going to come into the hospital and that they would start to rely on the hospitalists to manage the patients for them…. I think the technology accelerated that transition.” (Registered Nurse) A physician at a different site noted, “Yes, I think the introduction of CPOE corresponded to the rapid disappearance of primary care doctors from our hospital. I won’t say that in some cases, CPOE didn’t encourage the exodus. So, rather than have to learn something new, I think some of our primary care doctors used it as yet another excuse to say, ‘I’m not going to the hospital any longer. I lose money. I lose efficiency. I know that care won’t be as good with hospitalists, but I’m doing this.’ So I think it probably did catalyze the departure of many of our primary care doctors.”

## Discussion

In response to the availability of multi-million dollar incentives for meaningful use of health information technology to hospitals through HITECH as well other market forces under health care reform, hospitals in the United States are increasingly adopting CPOE. This multi-method study observed clinical activities and captured the experiences of physicians, nurses, pharmacists and administrators at five community hospitals in Massachusetts that had recently adopted CPOE. Our inter-disciplinary research team included physicians, a nurse and a pharmacist, allowing us to bring varied clinical perspectives to the analyses of the broad array of attitudes and experiences represented in the data. Analyses identified five domains that influenced the implementation process: governance, preparation, support, perceptions and consequences.

The findings of our study resonate with previous research on CPOE adoption and implementation in a variety of settings. Poon and colleagues carried out a mixed-methods study of barriers to CPOE of a diverse sample of hospitals across the US and identified several strategies, including strong leadership and engagement of clinicians, that were integral to successful implementation [17]. Ash, Sittig and colleagues have emphasized both anticipated and unintended consequences of implementation, [18,19] themes echoed by participants in our study.

Considerable literature has emerged regarding the organizational factors and theoretical frameworks for the implementation of health IT [20-22]. Systematic reviews have shown that individuals’ perceptions of new technology, its ease of use, and its impact on the time they spend to carry out their day-to-day tasks are among the key factors that facilitate adoption, while technical concerns, lack of training and negative attitudes of the users frequently manifest as obstacles to successful implementation [23-27].

Within the context of the existing literature, our results paint a picture of CPOE implementation in community hospitals that can be viewed as five “lessons learned” that may provide a new framework other community hospitals embarking on CPOE adoption.

### Lesson 1: governance matters

In any complex transformation, management of change requires vision, planning, leadership, and acceptance. The most salient theme we encountered throughout the course of interviews at all five hospitals was that the entity that manages this change needs to have representation from among the staff members, who will bear the greatest burden of the transformation, namely the physicians, nurses, pharmacists and other front-line staff. While the implementation of CPOE in a hospital remains at its core a project that must be managed under the auspices of an information systems department, clinical involvement is essential and clinicians and staff must have a seat at the table. This representation allows all stakeholders to feel valued and to have direct input in making key decisions which have a major impact on care delivery, especially with respect to efficiency. Furthermore, it facilitates communication, because the clinical leaders who serve on the governing board or committee serve as the liaison to their colleagues on the front lines.

### Lesson 2: preparation and advance planning

A key focus of our interviews was how the hospitals prepared nurses, physicians, pharmacists and other staff for the implementation of CPOE, a process that required months of preparation. We recognize that the planning that each hospital must undertake in advance of CPOE implementation – from needs assessment, to vendor selection, to hardware and software purchase and installation, to training – is a process that often takes two years and sometimes longer. With respect to preparing the staff, though, our study suggests that a “one-size-fits-all” approach to training and education is not likely to succeed. Participants emphasized the importance of understanding how much training each individual needs, recognizing that more experienced staff and clinicians, who may be older and less adroit with computers than younger individuals, may need more intensive preparation and training that begins with the basics of computer operation.

### Lesson 3: support at the elbow

Although formal training in computer skills and in the use of the CPOE system prior to implementation was regarded as necessary preparation, most participants, regardless of their professional role, noted the value of real-time, live, in-person support – usually by a peer superuser – in the weeks following “go-live.” Having a colleague “at the elbow” likely serves multiple functions. On the surface, the superuser assists the user in the immediate need of entering the order that is required for patient care. At a deeper level, though, this peer superuser is able to model facility with using the system, provide the user with encouragement and positive reinforcement, and commiserate with the user about the challenges inherent in any transition. Peer support seems to be an essential element of successful implementation. All the hospitals in this study trained their own superusers from among local peers, rather than employing superusers from an outside consulting firm. Whether outside experts are more or less effective than local peers is unknown. However, what is important to consider is whether the deployment of local peers as superusers will deplete the existing pool of clinical staff – particularly in nursing – creating a strain on the remaining staff.

### Lesson 4: managing perceptions

Not surprisingly, anxiety and fear of change were common across all five hospitals and pervasive among nurses and physicians, though not so among pharmacists. In some cases, especially among the older staff, the anxiety and fear seemed related not only to the concept of change but also to real concerns about computer proficiency. Awareness of these attitudes and prevailing moods is an important first step for those planning CPOE implementation. How best to manage the perceptions seemed to vary, and likely depends in part on the underlying culture of the institution (or unit within the institution). Strong leadership was a common theme that emerged, as was the importance of identifying and supporting a “champion” among each stakeholder group (e.g., physician champion, nurse champion, pharmacy champion) who can serve as liaison for the stakeholders, ensure that their concerns are addressed by institutional leadership, and provide reassurance to his or her peers.

### Lesson 5: truth or consequences

Participants in this study all had the benefit of hindsight in offering their perspectives, and with that hindsight came the opportunity to assess the consequences of implementing CPOE in their institutions. No interviewee expressed a preference to return to the pre-CPOE era. It was clear that the implementation process was not without some painful consequences, notably the apparent acceleration of retirement for some older nurses and physicians, and the transition of primary care doctors to using a hospitalist system. Moreover, many participants recognized that the implementation of CPOE was not a panacea for their hospital’s struggles to improve quality and safety. For every benefit of CPOE mentioned, an equally vocal chorus of concern about the unintended adverse consequences of CPOE and its risks to patient safety was expressed. A recent Institute of Medicine report addressed some of the issues around the new problems that HIT can create, and suggested approaches for dealing with them [28]. Hospitals that recognize these potential consequences at the outset will reap several benefits. First, they may be able to discuss the potential for adverse consequences with stakeholders, leading to collaborative approaches to bypass or ameliorate them. Second, they may be better positioned to recognize other unexpected consequences that are almost sure to emerge idiosyncratically at each hospital. Third, awareness of the potential for adverse consequences allows leaders to set more realistic expectations for stakeholders, which may ease the transformation process.

These findings have several implications. Strong financial incentives have been implemented to incent organizations to adopt CPOE, and organizations should be considering findings such as the above when they implement. The US Office of the National Coordinator for Health Information Technology is trying to make it easy for organizations to share approaches that have improved their likelihood of success. However, it is also important for organizations to be able to share problems that they identify, including software issues. Many vendors have contracts that attempt to preclude providers from sharing issues that they identify, including screenshots which appear to have caused errors. The recent Institute of Medicine report on this area suggests that vendors should not be allowed to restrict dissemination of potential patient safety issues. Whether or not the meaningful use incentives deliver the desired benefits in hospitals will depend on the extent to which organizations can assimilate lessons such as the ones identified in this study. Finally, this study raises several key topics for future study, including the extent to which CPOE and other HIT implementation efforts are accelerating the retirement or career transitions of nurses, physicians and other health care professionals.

### Limitations

As with any qualitative research, the validity of the results of this study should be considered in the context of established criteria, such as the Consolidated criteria for reporting qualitative research (COREQ), [29] which identifies three domains of criteria: the study team and reflexivity; study design; analysis and findings. While this study satisfies most of the 32 COREQ criteria, we note a few important limitations. In terms of the study team and reflexivity, we recognize that, despite attempts to remain objective, our own biases and experiences in research and health care delivery may have influenced our conduct of the interviews and observations, as well as the analysis of the data. With respect to the study design, we did not return transcripts to the interviewees for comment or correction. In our analyses, we did not formally conduct “participant checking” (i.e., asking interviewees to provide feedback on the findings); however, we did present our findings to the leadership of each hospital, who provided feedback that further guided our interpretation of the findings.

Another useful rubric for assessing qualitative research is to consider the “trustworthiness” of the research, [30] frequently operationalized as four constructs: a) credibility; b) transferability; c) dependability; and d) confirmability. To ensure credibility, we employed a variety of techniques, including the use multiple data collection methods (observation and in-depth interview), frequent meetings among the investigators to review and verify findings, and assessment of the findings in the context of prior literature. Transferability refers to the external validity or generalizability of the study. We conducted a qualitative study among a select group of five community hospitals that, for the most part, completed successful CPOE implementations in the years preceding our observations and interviews. We are able to report only on the attitudes and behaviors that existed at these hospitals – as perceived by the stakeholders themselves. Whether similar perceptions and observed experiences and outcomes would occur in other settings remains to be tested. Nevertheless, insofar as these community hospitals are representative of hospitals of similar size and composition across the country, the lessons learned in these five institutions may be instructive for other hospitals embarking on CPOE implementation in the near future. The dependability of this study is reflected in the high fidelity between the research proposed (in a written application to the funder) and that which was conducted, as reported here. Finally, confirmability of the study was reflected in our presentation of the findings to the leadership of each study hospital and their general agreement with the findings; however, as noted above, we did not conduct participant checking with each of the interviewees.

## Conclusions

Change is never easy, and change that involves transformation of workflow in the context of caring for hospitalized patients presents special challenges to all those involved. Those implementing CPOE in a community hospital may benefit from considering the concepts of governance, preparation, support, perceptions and consequences in their project design, so as to maximize the effectiveness of the implementation with the least adverse impact on all stakeholders.

## Abbreviations

CPOE: Computerized provider order entry; HITECH: Health information technology for economic and clinical health.

## Competing interests

Steven R. Simon, Carol Keohane, Mary Amato, Michael Coffey, Bismarck Cadet and Eyal Zimlichman have no competing interest. David W. Bates receives royalties from Medicalis, a company which has developed radiology order entry applications.

## Authors’ contributions

SRS designed the study, created the observation guide and in-depth interview questionnaire, conducted interviews, analyzed the data, and drafted the manuscript. CAK contributed to the design of the study, coordinated the study, conducted observations, and participated in the analysis of the data. MA conducted observations and interviews and participated in the analysis of the data. MC and BC contributed to the design of the study, assisted in recruitment of study participants, and participated in the analysis of the data. EZ conducted interviews and participated in the analysis of the data. DWB contributed to the design and coordination of the study, obtained funding, and participated in the drafting of the manuscript. All authors read and approved the final manuscript.

## Pre-publication history

The pre-publication history for this paper can be accessed here:

http://www.biomedcentral.com/1472-6947/13/67/prepub
